# In vitro assessment of the role of air spray on pulp temperature changes for preheated resin composite Class V restorations in lower incisor

**DOI:** 10.1007/s10266-026-01324-4

**Published:** 2026-02-19

**Authors:** Vinicius Borges Oliveira, Juliana Anany Gonzales Guarneri, Milena Ferreira Machado, Lucas Arrais Campos, Cesar Augusto Galvão Arrais

**Affiliations:** 1 Department of Restorative Dentistry, State University of Ponta Grossa, General Carlos Cavalcanti St., 4748, Uvaranas, Ponta Grossa, Parana 84030-900 Brazil; 2 Institute of Dentistry, School of Medicine, Faculty of Health Sciences, University of Eastern Finland, Yliopistonrinne 3, Canthia Building, Staircase B, P.O. Box 1627, 70211 Kuopio, Finland; 3 Faculty of Medicine and Health Technology, Tampere University, Finn-Medi 1, Arvo Ylpön katu 6, 33521 Tampere, Finland

**Keywords:** Dental pulp, Temperature, Composite resin, Dental curing light, Dental restoration, Permanent

## Abstract

To evaluate the effect of 15- and 30-psi air spray on in vitro pulp temperature (PT) during light-curing of buccal Class V restorations in lower incisors using preheated resin composites (RBCs). After ethical approval, a Class V cavity was prepared in a sound lower central incisor. A T-type thermocouple was inserted into the pulp chamber and connected to a temperature measurement system (Thermes WiFi, Physitemp). Simulated pulpal fluid flow was maintained at a rate of 0.001 mL/min. Real-time PT and working time were recorded during restoration with Viscalor Bulk-fill (VOCO) and Filtek One Bulk-fill (Solventum) preheated to 68 °C. Air spray (15 or 30 psi) was directed to the lingual surface, starting 3 s before and maintained throughout light-curing (*n* = 9). Vickers hardness (VHN) was measured at the middle and inner surfaces near the top and bottom of each specimen. PT and VHN data were analyzed using nonparametric mixed ANOVA, and working time was analyzed using nonparametric two-way ANOVA, both followed by Bonferroni’s post hoc tests (*α* = 0.05). Light-curing of preheated RBCs significantly increased PT (42.5 ± 1.3 °C; *p* < 0.001). Air spray at 15 and 30 psi reduced PT values (28.4 ± 1.3 °C and 26.8 ± 1.8 °C, respectively; *p* < 0.001) without affecting VHN values. Air spray during light-curing effectively minimized pulp temperature rise during restorative procedures using preheated RBCs on lower incisors.

## Introduction

Light-cured resin-based composites (RBCs) are widely used to restore both the aesthetic appearance and mechanical properties compatible with tooth structure. During this procedure, light exposure and the exothermic polymerization of these materials can transfer heat to the tooth [1, 2]. Depending on the characteristics of the light-curing unit (LCU) [3], irradiance and radiant exposure values [4, 5], remaining dentin thickness [6], fluid movement in dentinal tubules [7, 8], and the extent and type of cavity preparation [8–11], the pulp temperature (PT) may rise to levels considered harmful (above 43 °C) [7, 12, 13]. As a result, the heat generated during restorative procedures has raised significant concern among clinicians and researchers.

Despite these concerns, some manufacturers have recommended the use of preheated RBCs [14, 15]. Briefly, the RBC material is heated to 54–68 °C in a heating device before insertion into the cavity preparation [16]. Preheating enhances mechanical and physical properties, such as hardness and degree of conversion [17–22], even with shorter light exposure times [19, 20], and may also increase shear bond strength to dentin [23]. Although these materials reach high temperatures, studies have shown that preheated RBCs can be safely used in molars and premolars, as the heat transferred to the tooth contributes little to the rise in pulp temperature [24, 25]. However, these findings are limited to posterior teeth; there is no data on anterior teeth, such as lower incisors, which have a lower crown volume and thinner remaining dentin [26]. Given these anatomical differences, the heat transferred to a crown with a lower volume, such as incisors, may cause a higher PT increase than that observed in premolars or molars [8]. Consequently, the pulp of lower incisors is more prone to thermal injury than that of molars [27]. Nevertheless, there is no information available in the literature regarding the influence of preheated composites on the temperature changes within the pulp chamber of lower incisors.

To reduce the heat transmitted to the pulp, some authors have proposed applying an air spray to the tooth during light curing [28]. Briefly, an air spray is applied to the lingual surface of the tooth 3 s before light activation and is maintained during light exposure [28]. Consequently, as the temperature of the surrounding tooth structure drops, higher thermal energy is required to heat the enamel and dentin [28]. Therefore, such a procedure not only prevents the PT from rising but also reduces pulp temperature in premolars [28]. On the other hand, as the temperature of the surrounding tooth structure decreases, a greater temperature gradient is created between the preheated RBC and the tooth structure [28, 29], leading to heat loss from the RBC toward the tooth structure to reach thermal balance [28, 29]. Since lower initial temperatures of RBCs can negatively affect the degree of conversion and mechanical properties of these materials [30], it is crucial to investigate whether such cooling approaches might compromise the mechanical characteristics of preheated RBCs. However, to date, no information is available regarding the temperature rise in the pulp chamber of lower incisors during restorative procedures on buccal Class V cavities using preheated RBCs, nor regarding the effects of air spray on temperature within the pulp chamber or on the mechanical properties of these products.

Thus, this study aimed to evaluate the in vitro PT increase during the restoration of a buccal Class V preparation in a human lower central incisor using preheated RBCs at 68 °C, as well as the influence of air spray applied during the light-curing of preheated RBCs on the PT increase within the pulp chamber and on the mechanical properties of the preheated RBCs. The null hypotheses tested were:

1. Light-curing preheated RBCs at 68 °C in class V preparations in a lower central incisor does not cause a PT increase above values considered harmful to the pulp (43 °C);
2. Applying an air spray during light-curing does not prevent the temperature increase;
3. Using the air spray does not affect the hardness values of preheated RBCs.

## Materials and methods

### Spectral irradiance analysis of the LCU

To ensure the consistency of the light-curing unit (LCU) and to validate its output, the spectral irradiance of the LCU (lot no: #932125044020, Elipar Deep Cure-L, Solventum, St Paul, MN, USA) was measured using a laboratory-grade spectroradiometer (Flame, Ocean Optics, Dunedin, FL, USA) connected to a 15.24 cm integrating sphere (Labsphere, North Sutton, NH, USA), previously calibrated with a NIST-traceable light source. The LCU tip was positioned at the entrance of the integrating sphere to capture all emitted light (*n* = 5). The emission spectrum (425–490 nm) was recorded for the LCU using SpectraSuite v2.0.146 (Ocean Optics), which also provided the total emitted power over this wavelength range. The area of the distal end of the light guide was calculated using a digital caliper (Model 100.174BL, Digimess, São Paulo, SP, Brazil) with 2.2 × digital magnification (Magnifier, Bluelight Nova App, AppBrain, Utrecht, Netherlands). The integrated spectral power was divided by that area to determine the radiant emittance (mW/cm^2^).

### Tooth preparation

**Fig. 1:**
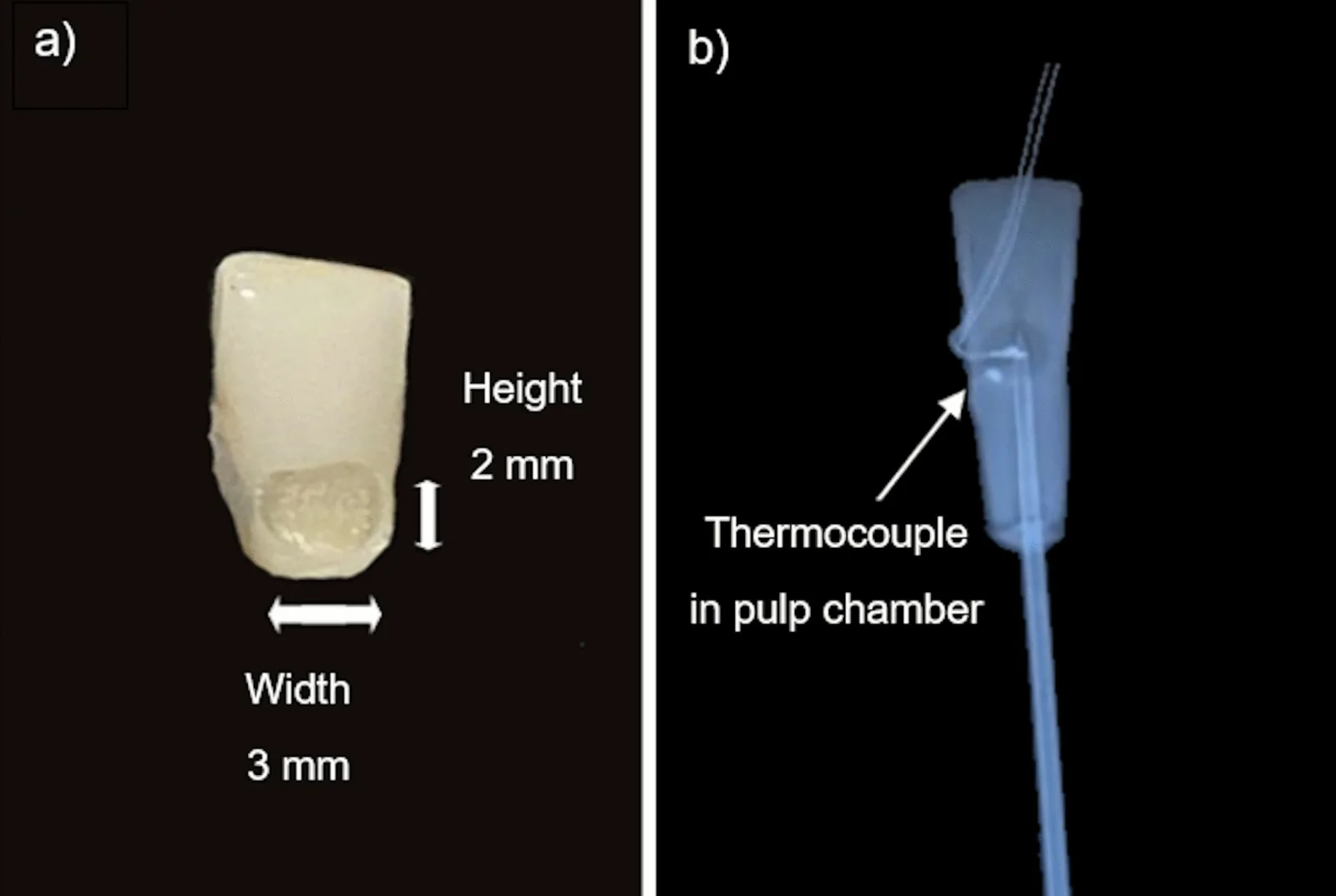
**a** Buccal Class V preparation on a lower incisor. **b** X-ray image of the Lower Central Incisor with the arrow indicating the thermocouple and the needle inside the pulp chamber

A sound mandibular central incisor was used, obtained from the local Human Tooth Bank. The specimen collection was approved by the local Research Ethics Committee (REC) (protocol no. 5.510.316/2022), and the informed consent form for tooth donation was obtained and archived by the Human Tooth Bank, following the institution’s protocol. A Class V preparation was made on the buccal surface near the cement-enamel junction using a medium-grit diamond bur (FG 2131, KG Sorensen, Cotia, São Paulo, Brazil). The axial wall was approximately 1 mm thick, the cavity width was approximately 3 mm, and the cavity height was approximately 2 mm (Fig. 1a, b), while the preparation depth was approximately 1.5 mm. The dimensions were verified using a North Carolina periodontal probe No. 15 (Hu-Friedy^®^, Chicago, IL, USA).

### In vitro measurements of pulp temperature increase

A single experienced operator performed all procedures to ensure methodological consistency throughout the study. Simple randomization was used to allocate experimental groups. The root was sectioned 4 mm below the cementoenamel junction, and cleaning and enlargement of the root canals were carried out using Gates Glidden burs N. 3 and N. 4 (DentsplySirona, York, Pennsylvania, USA). Subsequently, a perforation was made on the proximal surface, approximately at the cement-enamel junction. A T-type thermocouple was inserted into the pulp chamber through the orifice (Fig. 1b). Afterwards, the orifice was sealed with flowable RBC (Opallis Flow, FGM, Joinville, SC, Brazil), which was light-cured as recommended by the manufacturer. The thermocouple wire was insulated with a blue Rubber Dam (Sanctuary, K-Dent, Joinville, SC, Brazil), adhesive tape (Scotch™, Solventum, St. Paul, MN, USA), and PU foam (Unipega, Florianopolis, SC, Brazil).

**Fig. 2:**
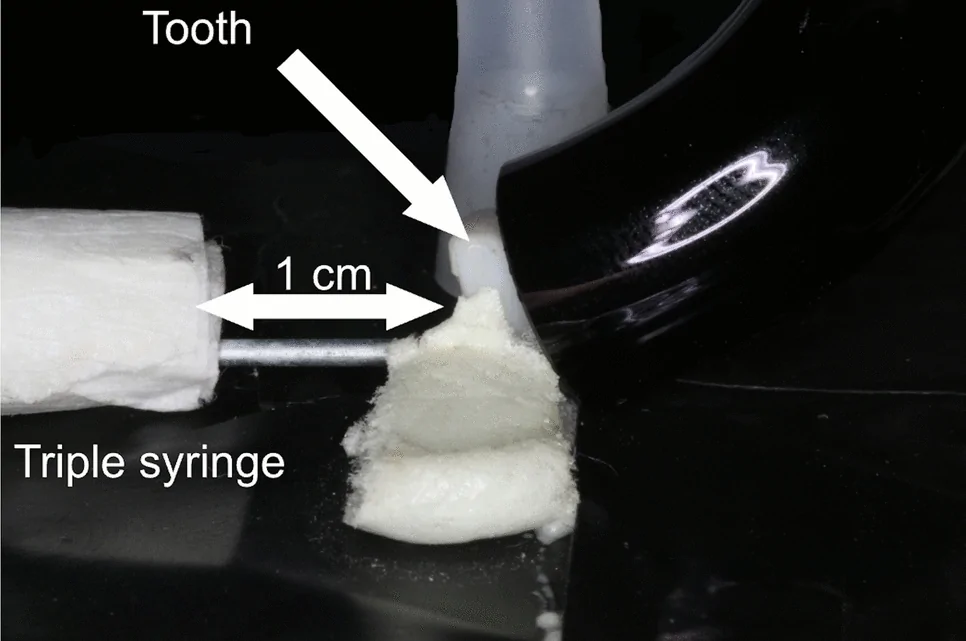
The LCU tip is positioned on the buccal surface of the tooth, as indicated by the arrow, while the triple syringe tip is located on the lingual surface, 1 cm away

**Fig. 3:**
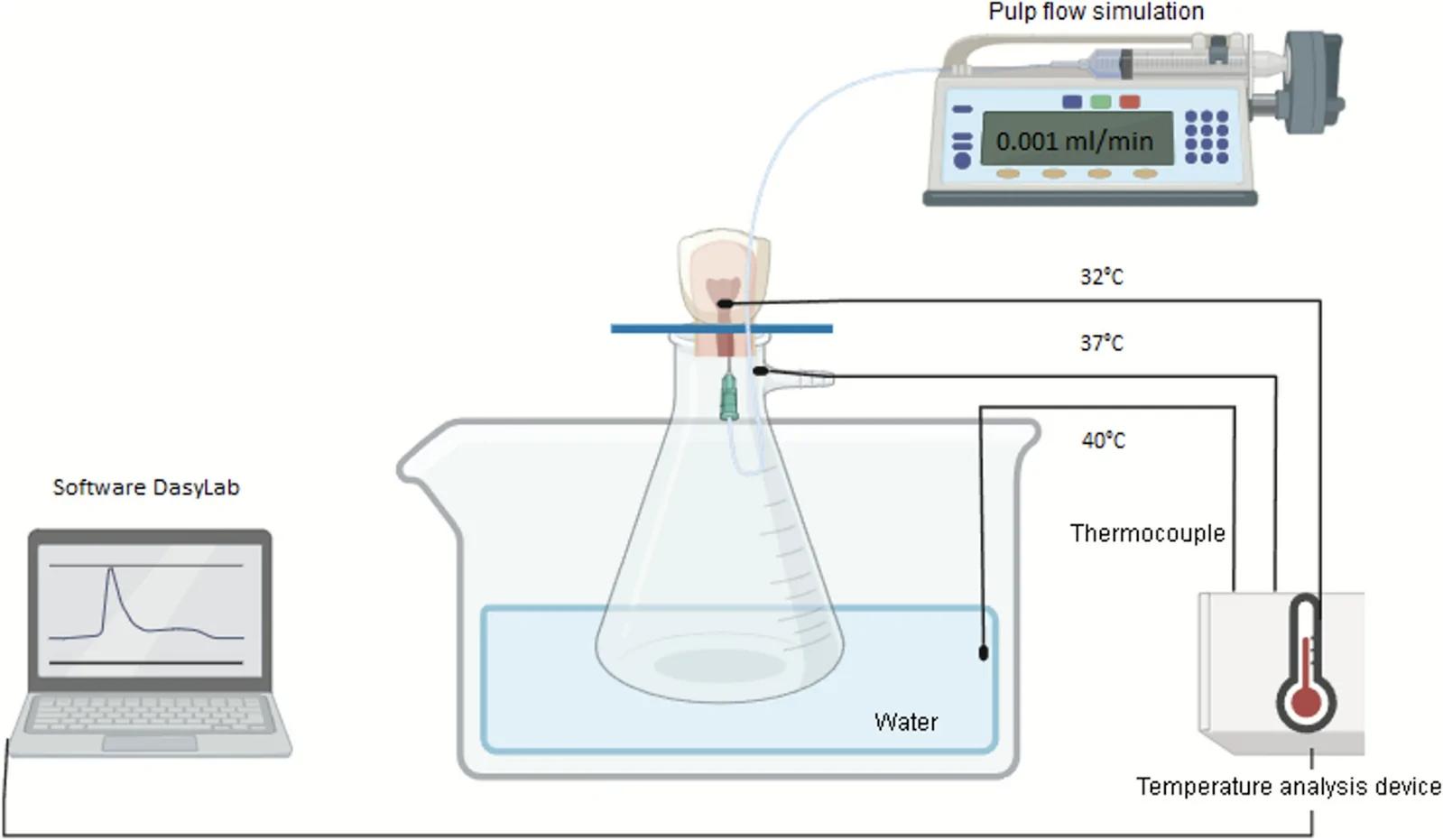
Schematic diagram of the method for analyzing pulp temperature

The tooth was fixed on an acrylic plate with a central opening, simulating rubber dam isolation [11] (Fig. 2). A tube was connected at one end to the root and at the other end to an infusion pump (RS700 Syringe Pump, RZ Veterinary Equipment, São Paulo, SP, Brazil) to simulate pulpal flow (Fig. 3). The acrylic/tooth assembly was placed in the opening of a Kitasato flask filled with heated water maintained at a controlled temperature of 37 °C, with the flask partially immersed in a water bath at 40 °C. Therefore, a constant water flow of 32 °C was maintained in the pulp chamber, as this value is widely used in in vitro pulp temperature simulation studies [11, 31, 32] and approximates the average intra-pulpal temperature of a vital tooth in a clinical setting after an etch-and-rinse adhesive system is applied [33].

The infusion pump was programmed to maintain a pulpal flow of approximately 0.001 mL/min, similar to the pulp blood flow [34]. The rate was calculated using existing literature. The average volume of pulp chamber in a human, lower incisor is approximately 6.1 mm^3^ (0.0061 cc) [26]. Assuming a pulp tissue density roughly equal to water (1 g/mL), this gives an estimated pulp mass of 0.0061 g. The reported pulpal flow rate in dogs is 33.32 mL/min for each 100 g of tissue [35]. Applying this rate to the mass of pulpal tissue calculated in a lower incisor yields a flow rate of approximately 2.03 μL/min. To account for the known ~ 25% reduction in pulpal blood flow in human teeth due to the vasoconstrictive effect of local anesthetics [36], the rate was adjusted to 0.00152 mL/min (1.52 μL/min). The closest achievable setting on the available infusion pump to this calculated clinical rate was 0.001 mL/min (1.0 μL/min), which was used for all experiments.

Real-time temperature analysis was measured inside the pulp chamber at 0.5 s intervals using the type T thermocouple connected to a temperature measurement system (Thermes WiFi, Physitemp, New Jersey, USA) (Fig. 3). The room temperature was recorded daily during all analyses using a digital thermometer (7665.02.0.00, Incoterm, China), with an average temperature ranging from 22 to 24 °C.

A thin layer of water-soluble lubricant (K-Y gel, Johnson & Johnson, New Brunswick, NJ, USA) was applied inside the cavity using a microbrush (FGM, Joinville, SC, Brazil). A piece of dental floss was inserted into the cavity preparation to allow an easy removal of the cured RBC [37]. Although the thermal conductivity of the water-soluble lubricant is not available in the manufacturer’s technical data, the product can be characterized as a water-based hydrogel containing approximately 80–85% water and 10–15% polyols (glycerin and propylene glycol). Because the thermal conductivity of aqueous mixtures decreases with the proportion of polyols (water ≈ 0.60 W m⁻^1^ K⁻^1^; glycerin ≈ 0.29 W m⁻^1^ K⁻^1^; propylene glycol ≈ 0.21 W m⁻^1^ K⁻^1^) [38–40], the conductivity of K-Y Gel was estimated by composition-weighted averaging to be approximately 0.45–0.50 W m⁻^1^ K⁻^1^ (estimated from values reported for aqueous polyol solutions at 25 °C). For comparison, the thermal conductivity of an adhesive system can be assumed to range from 0.15 to 0.25 W m⁻^1^ K⁻^1^, similar to that of other polymeric materials with low filler content [41]. Dentin, in contrast, has a conductivity around 0.45–0.50 W m⁻^1^ K⁻^1^ [42, 43]. Considering a 20-µm thick bonding layer and a 1-mm thick remaining dentin wall, the thermal resistance of dentin (≈ 1.8 × 10⁻^3^ K m^2^/W) is one to two orders of magnitude higher than that of either K-Y Gel (≈ 4 × 10⁻^5^ K m^2^/W) or the adhesive (≈ 1 × 10⁻^4^ K m^2^/W). Consequently, the interface accounts for only 2–5% of the total thermal resistance, indicating that the interfacial medium's effect on pulpal temperature rise is minimal under these conditions [38–40, 44].

Two bulk-fill RBCs were evaluated (Table 1): a thermoviscous resin composite, VisCalor Bulk (VCBF, lot #2206543, shade A2; VOCO, Cuxhaven, Germany), which requires preheating before application [45], and Filtek One Bulk-fill (FOBF, lot #9291076, shade A2; Solventum, St. Paul, MN, USA), a material that does not require preheating but can be optionally preheated according to the manufacturer’s recommendations [46]. The Caps Warmer device (VOCO, Cuxhaven, Germany) was heated for 15–20 min before the cavity was restored. Capsules of VCBF and FOBF RBCs were placed in the Dispenser Caps and were then placed in the Caps Warmer heating device. The manufacturers recommended heating times of 5 min for FOBF and 3 min for VCBF to reach 68°C. After heating, each capsule was removed from the device, and the Class V cavity was filled with the preheated RBCs using the Dispenser Caps. Both RBCs were inserted into the prepared cavity in a single increment, according to the experimental groups, and light-cured on the buccal surface for 20 s (Elipar™DeepCure-L, Solventum), as recommended by the manufacturer. Irradiance was continuously measured using a handheld radiometer (Bluephase Metter II, Ivoclar, Schaan, Liechtenstein).

**Table 1:** Materials used in the study and their respective compositions as indicated by the manufacturer

Material	Composition
Filtek one bulk-fill (Solventum)	AUDMA (10–20%); DDDMA (< 10%); UDMA (1–10%); silane-treated ceramic (60–70%); silane-treated silica (1–10%); ytterbium fluoride (YbF3) (1–10%); silane-treated zirconia (< 5%); water (< 5%)
Viscalor bulk (VOCO)	Bis-GMA (10–25%); aliphatic dimethacrylates (10–25%); nanohybrid inorganic filler (83%)

*AUDMA* aromatic urethane dimethacrylate, *DDMA* 1,10-decanediol dimethacrylate, *Bis-GMA* bisphenol A-glycidyl methacrylate

Six experimental groups were defined by combining the composite used (VCBF or FOBF) with the air-spray condition (no air, 15 psi, or 30 psi). The control groups (VCBF or FOBF without air) represented preheated RBCs cured without airflow. When 15- or 30-psi air spray was applied, the tip of the triple syringe (model Premium; Gnatus Equipamentos Médico-Odontológicos Ltda., Ribeirão Preto, SP, Brazil) was positioned 1 cm away from the lingual surface (Fig. 2), while the LCU tip was positioned on the buccal surface. The air spray started 3 s before and during light-curing as previously reported [28]. Air pressure was controlled by a pressure regulator (AER2000N, Fluir pneumática, Mirassol, Brazil) and set to the standard air pressure on the chair. The internal diameter of the triple-syringe nozzle was approximately 0.8 mm, and the air spray was applied at room temperature. Based on the upstream pressures used in this study (15 and 30 psi) and the atmospheric downstream pressure, the flow conditions satisfied the criterion for choked compressible flow. Under choked-flow conditions, the air exits the nozzle at sonic velocity, which is determined solely by the thermodynamic properties of air and the ambient temperature. Therefore, the airflow velocity at the nozzle outlet was estimated to be approximately 343 m/s for both pressures. Nine restorative procedures were performed in each experimental group (*n* = 9).

### Vickers hardness measurement (VHN)

After light-curing of the RBCs, the specimens were carefully removed from the Class V cavity. Subsequently, the specimens were placed in PVC tubes using Flat No. 7 Wax (Asfer Indústria Química, São Caetano do Sul, SP, Brazil) to ensure stable and uniform positioning during the embedding process. The specimens were then embedded in epoxy resin (SMC, Cesário Lange, SP, Brazil) within the PVC tubes and were stored in a controlled environment for 24 h. Afterwards, the specimens were abraded on the proximal surface (Arotec S/A Indústria e Comércio, Cotia, SP, Brazil) with silicon carbide abrasive papers with constant irrigation in decreasing grit sizes: 600, 1200, and 2400 respectively (401Q Wetordry™ Paper, 3M, Maplewood, MN, USA), for 10 s each, exposing and flattening the middle, inner specimen surface. The surface was polished with metallographic polishing cloths and 3- and 1-micron diamond pastes (Arotec S/A Indústria e Comércio, Cotia, SP, Brazil) for 30 s each. The cured specimens were placed in an ultrasonic bath (Cristófoli Equipamentos de Biossegurança LTDA., Campo Mourão, PR, Brazil) for 30 min. The polished, middle inner surfaces were positioned in the microhardness tester (SHIMADZU HMV-2T, Newage Testing Instruments, Southampton, PA, USA), and hardness was measured in triplicate on the top and bottom regions near the edge (distance of approximately 1.5 mm) of the RBC inner surface. Hardness was measured under a load of 200 g (1961 N) with a dwell time of 15 s.

### Temperature measurement of the preheated RBCs during working time

Given that previous studies reported a temperature drop in the preheated composite during handling [25], temperature measurements were also performed during heating and during transport to the Class V preparation. To measure the temperature range, a T-type thermocouple was inserted into the capsules of both RBCs. The T-type thermocouple was connected to a temperature measurement system (Thermes WiFi, Physitemp, New Jersey, USA), enabling real-time measurements at 0.5 s intervals.

Five repetitions (*n* = 5) were performed for each material. The time was counted, and the temperature was measured at two moments: (1) from the moment the RBC was removed from the Caps Warmer device until the start of insertion into the cavity (Moment 1); and (2) during the time spent placing and sculpting the RBC into the cavity (Moment 2).

### Statistical analysis

The minimum sample size was calculated using G*Power (version 3.1.97, Universität Düsseldorf, Germany) for each dependent variable based on data from a pilot study. A significance level of *α* = 0.05 and a statistical power (1 − *β*) of 90% (*β* = 0.10) were considered. The effect size was *f* = 0.26678, calculated from *η*^2^ = 0.076, indicating a medium effect and accounting for 7.6% of the total variance in the data. The RBC temperature, PT, working time, and VHN data were subjected to the Shapiro–Wilk test to verify the assumption of normality. The normal distribution and homogeneity of variances were evaluated using the Shapiro–Wilk test and the Levene’s test (*α* = 5%), respectively. At least one of the experimental groups did not meet the assumption of approximation to a normal distribution for both PT (Shapiro–Wilk test: *W* = 0.82, *p* = 0.023) and VHN (Shapiro–Wilk test: *W* = 0.66, *p* < 0.001). Therefore, for PT, a nonparametric three-way mixed ANOVA followed by Bonferroni’s post hoc test (*α* = 5%) was used, with measurement points (baseline and peak values) as the within-subjects factor and different RBCs and experimental groups as the between-subjects factors. For VHN, the analysis was conducted separately for each RBC. Therefore, comparisons between region (bottom or top, within-subjects factor) and experimental air spray groups (between-subjects factors) were performed using a non-parametric two-way mixed ANOVA. The working time data also did not meet the assumption of normality (Shapiro–Wilk test: *W* = 0.72, *p* = 0.02) and were compared across different RBCs and experimental air-spray groups using a nonparametric two-way ANOVA followed by Bonferroni’s post hoc test (*α* = 5%). Effect sizes (partial *η*^2^) with 95% confidence intervals were calculated for all main and interaction effects that reached statistical significance (*p* < 0.05). All statistical analyses were performed using SPSS 28 (IBM Corp., Armonk, NY, USA).

## Results

### Spectral irradiance

**Fig. 4:**
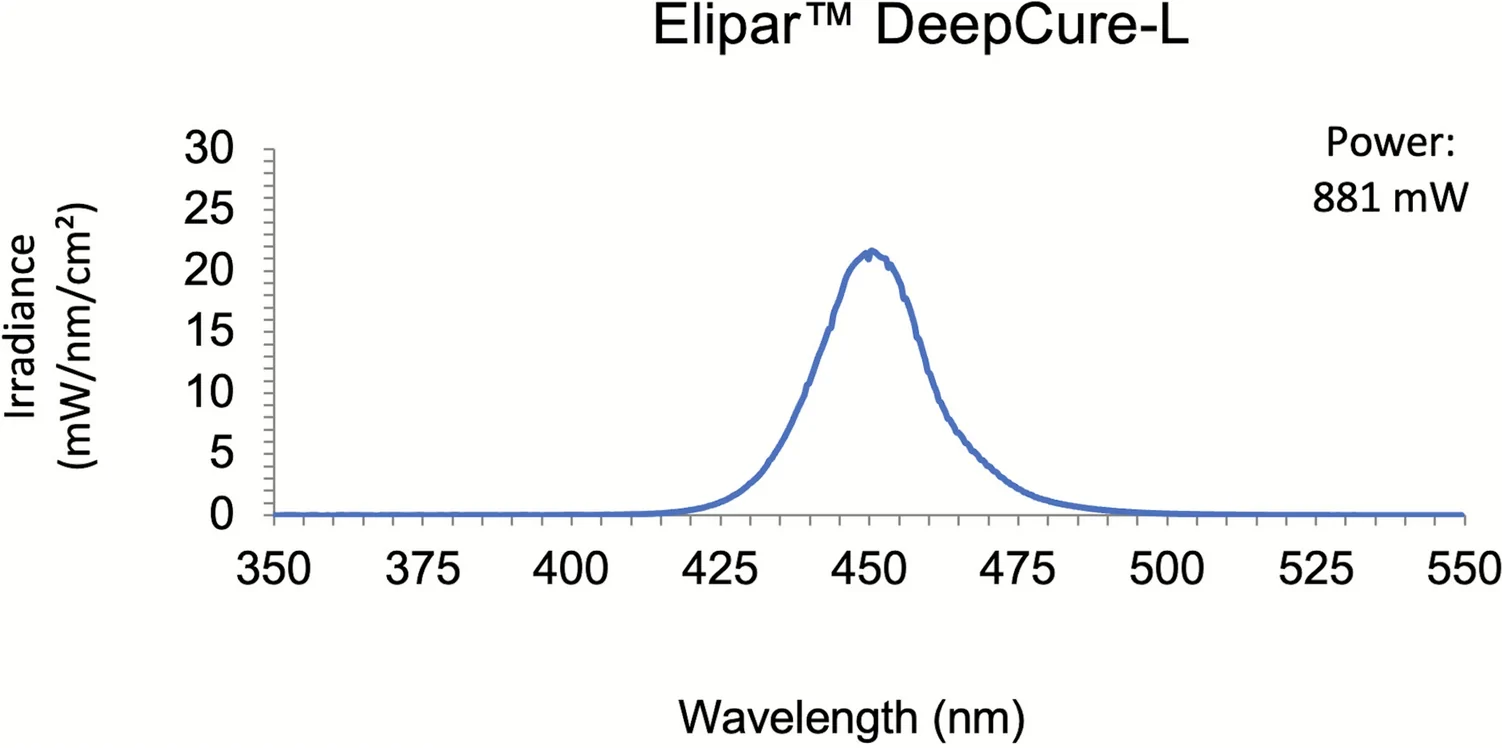
Power (mW), irradiance (mW/nm/cm^2^), and wavelength (nm) of the Elipar DeepCure-L LED Curing Light

In the present study, a monowave LED curing light, the Elipar™ DeepCure-L (Solventum), was used. The diameter of the curing tip was measured at 8.8 mm. The recorded power was 881 mW, and the irradiance was 1416 mW/cm^2^ (Fig. 4).

### In vitro measurements of pulp temperature increase

There was no interaction among the three independent variables (measurement points*RBC*air spray: *F* = 0.60, *p* = 0.562). The only significant interaction observed was between measurement points and experimental air spray groups (measurement points*air spray: *F* = 150.40, *p* < 0.001, *η*^2^_p_ [95%CI] = 0.848 [0.761–0.886]). Therefore, the results are expressed considering only the interaction of these two variables.

Table 2 shows the mean baseline and peak PT values for the experimental air-spray groups, regardless of RBC. When air spray was applied at 15 and 30 psi, the PT during light-curing decreased compared with that observed when no air spray was applied (Table 2). The 30-psi air spray reduced the peak temperature (26.8°C ± 1.8) to lower values than those observed in the 15-psi group (28.4°C ± 1.3) (*p* < 0.001).

**Table 2:** Mean (standard deviation) of baseline and peak pulp temperature (°C) according to the technique

Technique	Baseline	Peak
Without air	32.6 (0.3)^Aa^	42.5 (1.3)^Bc^
15 psi	32.6 (0.3)^Ba^	28.4 (1.3)^Ab^
30 psi	32.5 (0.3)^Ba^	26.8 (1.8)^Aa^

Means followed by different letters (uppercase letters within row; lowercase letters within column) are significantly different (preset *α* = 5%)

**Fig. 5:**
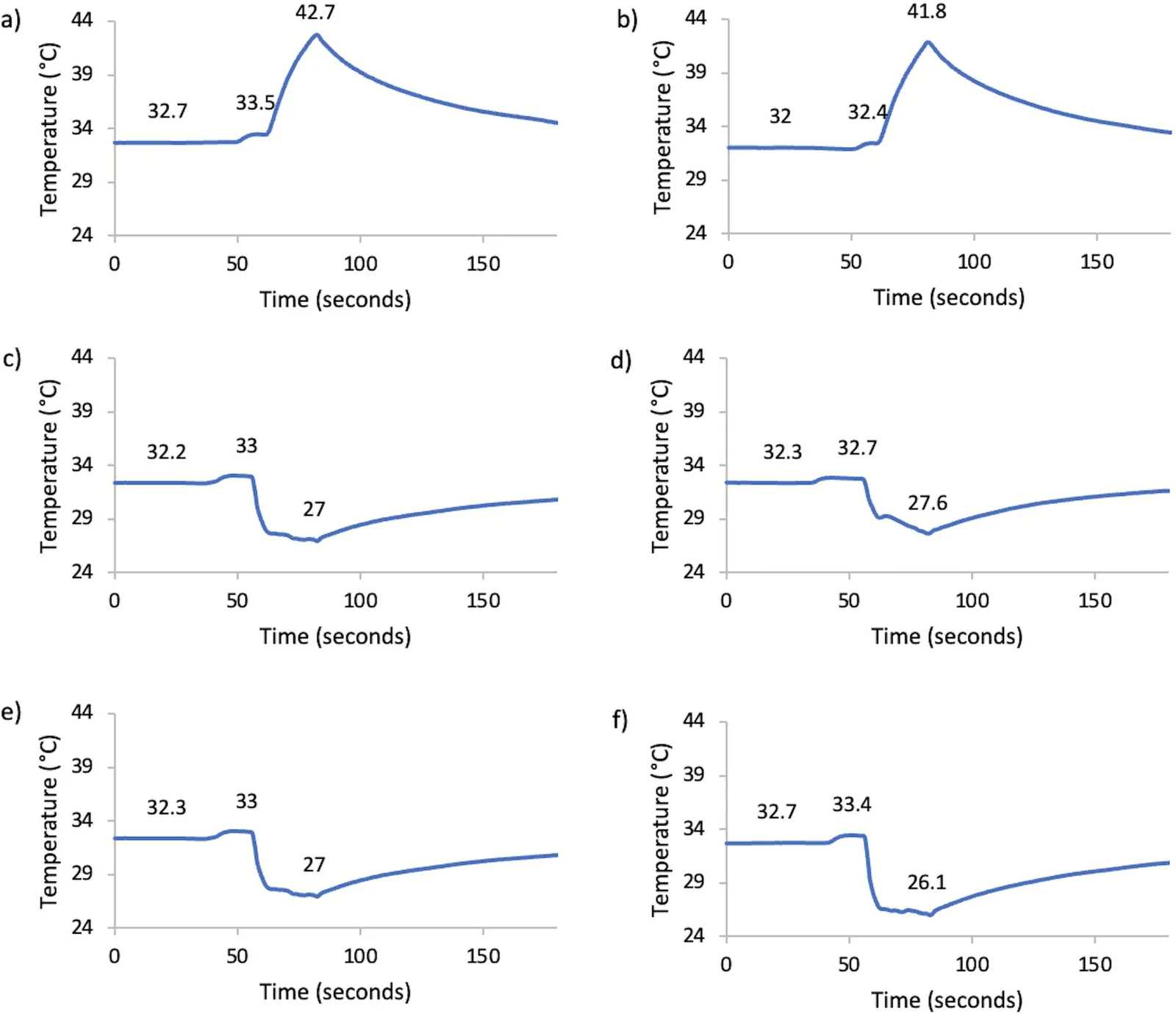
Representative real-time PT analysis of each experimental group. In sequence: **a** FOBF without air; **b** VCBF without air; **c** FOBF 15 psi; **d** VCBF 15 psi; **e** FOBF 30 psi; **f** VCBF 30 psi

The representative real-time temperature profiles within the pulp chamber are shown in Fig. 5. When the Class V preparation was filled with preheated RBC, a slight increase in PT values was noticed before the LCU light was turned on. When no air spray was applied, a rapid, significant increase in PT was observed as the LCU light was turned on in both the FOBF and VCBF (42.7 °C and 41.8°C) (Fig. 5a, b). When the air spray was applied, a quick drop in the PT values was observed during light-curing, followed by a gradual increase in PT values of both RBCs (27 °C, 27.6 °C, 27 °C, and 26.1 °C) (Fig. 5c–f).

### Vickers hardness (VHN) measurement

Table 3 shows the mean VHN values of the RBCs at the top and bottom regions for experimental air spray groups. For both FOBF and VCBF, no interaction was observed between the region and experimental air spray groups (*F* ≤ 1.42, *p* ≥ 0.261). For FOBF, VHN values were not affected by the air spray (*F* = 1.14, *p* = 0.337). A statistical difference was observed only for the region with the bottom exhibiting higher values than the top (*F* = 5.17, *p* = 0.032, *η*^2^_p_ [95%CI] = 0.177 [0.000–0.417]).

**Table 3:** The Vickers hardness values (VHN), standard deviation, and base/top ratio of the tested RBCs

	Technique	Surface	
		Bottom	Top
FOBF	Without air	72.2 (3.7)^Aa^	70.1 (0.7)^Ba^
	15 psi	71.4 (2.1)^Aa^	70.1 (0.9)^Ba^
	30 psi	71.6 (0.8)^Aa^	71.6 (0.7)^Ba^
VCBF	Without air	85.1 (4.6)^Aa^	84.3 (3.9)^Aa^
	15 psi	85.4 (2.8)^Aab^	86.0 (2.0)^Aab^
	30 psi	87.4 (0.8)^Ab^	86.8 (2.2)^Ab^

Means followed by different letters (uppercase letters within row; lowercase letters within column) are significantly different (preset *α* = 5%). No comparisons were made between products

For VCBF, a statistical difference was observed only between the experimental air spray groups (*F* = 4.60, *p* = 0.020, *η*^2^_p_ [95%CI] = 0.259 [0.004–0.462]) with the use of air spray resulting in the highest hardness values at both the top and bottom when compared to the control group (top = 86.8 ± 2.2 and bottom = 87.4 ± 0.8) (Table 3). VHN values of VCBF were not different between the top and bottom regions (*F* = 0.84, *p* = 0.367).

### Effect of working time on the temperature of preheated RBCs

**Fig. 6:**
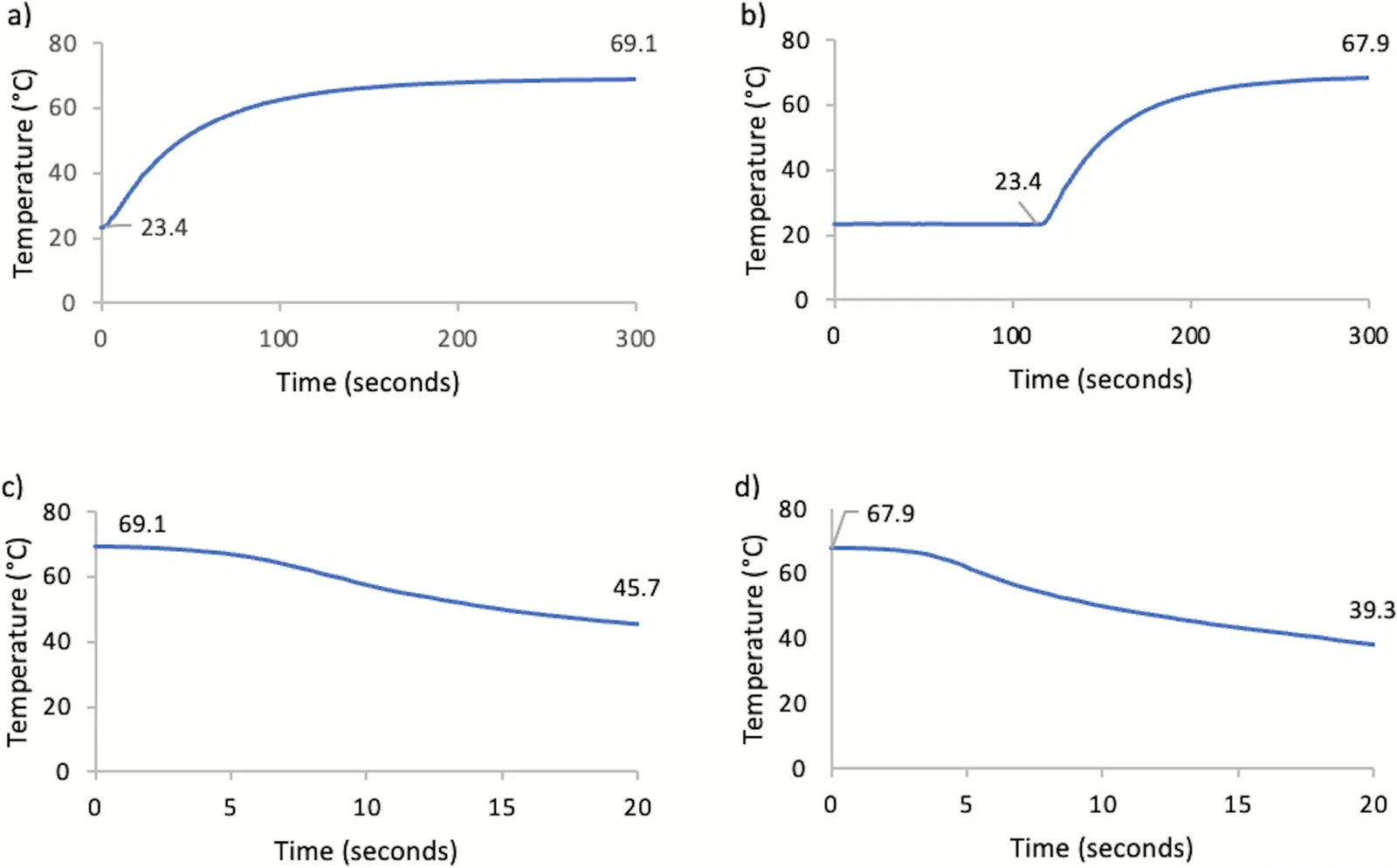
Representative, real-time temperature (°C) analysis of the RBCs inside the heating device (Caps Warmer). FOBF (**a**) and VCBF (**b**) temperature over a period of 5 min. The temperature drop in FOBF (**c**) and VCBF (**d**) during the working time (20 s) after their removal from the heating device

Both RBCs reached temperature values close to the expected preheating values of 68 °C (FOBF = 69.1 ± 0.03 °C and VCBF = 67.9 ± 0.6 °C) (Table 4, Fig. 6a, b). The temperature remained stable for a few seconds, then gradually decreased to 39.48 °C and 33.47 °C within 20 s for VCBF and FORBF, respectively (Table 4, Fig. 6c, d).

**Table 4:** Mean (standard deviation) of the temperature (°C) of the RBC inside the Caps Warmer device after heating and 20 s after removal from the device

Product	Caps warmer	After 20 s	Reduction	Percent reduction (%)
FOBF	69.13 (0.03)	45.72 (1.8)	-23.41 (1.8)	33.86
VCBF	67.91 (0.6)	39.39 (1.6)	-28.52 (2.1)	42.00

Table 5 shows the time required to deliver and sculpt the RBCs before light-curing. At Moment 1—defined as the time interval between the insertion of the RBCs into the cavity and the initiation of light-curing—no interaction between the independent variables was observed (*F* = 0.10, *p* = 0.889). Likewise, no statistically significant difference in working time was found between RBCs (*F* = 3.40, *p* = 0.072) or among techniques (*F* = 0.80, *p* = 0.451). At Moment 2, there was also no interaction between the variables (*F* = 0.50, *p* = 0.606). However, the time spent in the groups that included air-spray application was longer than that in the control group (*F* = 21.80, *p* < 0.001, *η*^2^_p_ [95%CI] = 0.447 [0.233–0.578]). No difference was observed between RBCs (*F* = 0.60, *p* = 0.444).

**Table 5:** Mean (standard deviation) of the time spent (s) from the removal of the RBC from the Caps Warmer (heater) until the beginning of the RBC insertion into the cavity (Moment 1) and from the RBC insertion into the cavity until light-curing starts (Moment 2), according to the product and technique

	Technique	FOBF	VCBF
Moment 1	Without air	14.6 (4.3)^Aa^	17.5 (7.1)^Aa^
	15 psi	14.7 (2.0)^Aa^	20.1 (8.0)^Aa^
	30 psi	14.7 (2.9)^Aa^	18.6 (8.4)^Aa^
Moment 2	Without air	11.4 (2.4)^Aa^	14.4 (7.8)^Aa^
	15 psi	17.8 (1.6)^Ba^	17.3 (3.0)^Ba^
	30 psi	16.7 (2.3)^Ba^	17.4 (2.0)^Ba^

Means followed by different letters (uppercase: within column; lowercase: within row) are significantly different (preset *α* = 5%). No comparison between moments was made

## Discussion

In this study, real-time PT was evaluated during a buccal Class V preparation in a lower central incisor restored with preheated bulk-fill RBCs. Although the mean peak temperature (42.5 ± 1.3 °C) did not reach 43 °C, some specimens exceeded this threshold. In other words, the chances of pulpal damage, such as plasma coagulation, fluid expansion in dentinal tubules, and tissue necrosis, should not be discarded [47]. Thus, the first null hypothesis, that light-curing of preheated VCBF and FOBF would not increase the PT to peak values considered harmful to the pulp (43 °C) [7], must be rejected. These findings highlight the need for cautious clinical use of preheated composites and for adopting strategies that minimize temperature rise during restorative procedures. The contact of preheated RBC with the cavity walls caused a slight increase in PT values (Fig. 5). These findings are consistent with those from previous studies [29], which also reported the heat transfer from the RBC to the tooth [29]. However, the heat transfer was insufficient to raise PT to harmful levels. This finding suggests that preheated RBCs were not the primary cause of the PT rise observed in the current study, indicating that the most significant risk to pulpal health occurs during light-curing [6, 48]. Although both RBCs reached temperatures close to those specified by the manufacturers after preheating, the rapid heat dissipation observed in both RBCs and the consequent temperature drop (33.86% and 42.00% decrease in FOPBF and VCBF, respectively) after their removal from the Caps Warmer device help explain this finding (Table 4 and Fig. 6).

To mitigate the heat generated during RBC light curing, an air spray was applied during curing. The results showed that PT values decreased during light-curing of both RBCs, falling below baseline when a 15- or 30-psi air spray was applied (Table 2). Therefore, the second null hypothesis, that the use of 15- and 30-psi air spray would not prevent an increase in PT values, was rejected. A similar decrease in PT was also observed in vivo [1] when buccal Class V preparations in premolars were subjected to a 28-psi air spray during light-curing. By cooling the cavity walls with an air spray, heat from the pulp is dissipated via thermal conduction to the cavity walls, reestablishing thermodynamic balance [1]. In addition, others have noted that air spray helps dissipate heat around exposed teeth [49].

Although some studies report that reducing temperature can impair monomer conversion [50, 51], air spray applied alongside photo-curing did not decrease the VHN values of preheated RBCs. Therefore, the temperature reduction caused by the air was not enough to hinder polymerization, so the third null hypothesis was not rejected. Curiously, the use of 30-psi air spray on VCBF increased the VHN values at the bottom and top. Although the clinical relevance of these differences is questionable, the results were unexpected, as the proposed technique does not address any factors that could improve monomer conversion. This phenomenon warrants further investigation.

In the current study, a buccal deep Class V preparation was performed on a lower incisor, leaving a 1-mm-thick remaining axial wall. Studies have demonstrated that dentin thickness plays a crucial role in both increasing and decreasing PT [6, 52]. For this reason, the current findings may not apply when the remaining axial dentin thickness is less than 1 mm. In addition, although the current study environment aimed to closely simulate a real clinical situation by simulating fluid flow at a physiological baseline PT, this study was conducted in vitro. Because this method cannot reproduce the complex physiological mechanisms underlying temperature control in pulp tissue, the current findings should be interpreted with caution.

The clinical relevance of this study lies in its direct implications for safe and effective restorative procedures. Preheating composite resins has become increasingly common due to documented benefits, including reduced viscosity, improved handling, and enhanced mechanical performance [18, 52, 53]. However, this practice raises concerns regarding potential intrapulpal temperature increases, particularly in teeth with reduced dentin thickness, such as mandibular central incisors, which are more susceptible to thermal transmission [54]. Understanding this balance between improved material performance and pulpal safety is essential for guiding evidence-based clinical decision-making. It is important to emphasize that this is an exploratory study based on a single tooth, which represents a significant methodological limitation. Although this model enabled strict control of internal variables, such as thermocouple positioning and standardized cavity geometry, it does not reflect natural biological variability and restricts the generalizability of the findings. Nevertheless, the evidence indicates that applying 15- or 30-psi air spray during light-curing is an effective protective strategy without affecting the composite's mechanical properties. Using the universally available dental air–water syringe, this approach reduced thermal stress and maintained intrapulpal temperature increases within safer thresholds, potentially preventing irreversible pulpal damage [12]. Therefore, this protocol should be clinically recommended as a preventive measure when using preheated bulk-fill resins in preparations close to the pulp. Future in vivo studies are essential to confirm these exploratory findings under actual clinical conditions.

## Conclusion

Within the limitations imposed by the methodology, it was possible to conclude that:

1. light-curing of preheated RBCs placed on buccal Class V preparations on the lower incisor may pose a risk to the pulp, as the PT rise can reach values considered potentially harmful;
2. air-spray applied during light-curing of preheated RBCs prevented the PT from rising during exposure to LCU light; and
3. using air-spray did not impair the polymerization of preheated RBCs as assessed by microhardness.

## Data Availability

The data that support the findings of this study are available from the authors upon reasonable request.
